# An Unwanted Association: The Threat to Papaya Crops by a Novel Potexvirus in Northwest Argentina

**DOI:** 10.3390/v14102297

**Published:** 2022-10-19

**Authors:** Dariel Cabrera Mederos, Humberto Debat, Carolina Torres, Orelvis Portal, Margarita Jaramillo Zapata, Verónica Trucco, Ceferino Flores, Claudio Ortiz, Alejandra Badaracco, Luis Acuña, Claudia Nome, Diego Quito-Avila, Nicolas Bejerman, Onias Castellanos Collazo, Aminael Sánchez-Rodríguez, Fabián Giolitti

**Affiliations:** 1 Unidad de Fitopatología y Modelización Agrícola, Consejo Nacional de Investigaciones Científicas y Técnicas, Córdoba X5020ICA, Argentina; 2 Instituto de Patología Vegetal “Ing. Agr. Sergio Fernando Nome”, Instituto Nacional de Tecnología Agropecuaria, Córdoba X5020ICA, Argentina; 3 Facultad de Farmacia y Bioquímica, Instituto de Investigaciones en Bacteriología y Virología Molecular, Universidad de Buenos Aires, Buenos Aires C1425FBQ, Argentina; 4 Consejo Nacional de Investigaciones Científicas y Técnicas, Buenos Aires C1425FBQ, Argentina; 5 Departamento de Biología, Facultad de Ciencias Agropecuarias, Universidad Central “Marta Abreu” de Las Villas, Santa Clara 54830, Cuba; 6 Centro de Investigaciones Agropecuarias, Facultad de Ciencias Agropecuarias, Universidad Central “Marta Abreu” de Las Villas, Santa Clara 54830, Cuba; 7 Universidad de San Pablo-T, Tucumán 4129, Argentina; 8 Estación Experimental Agropecuaria Yuto, Instituto Nacional de Tecnología Agropecuaria, Jujuy Y4518, Argentina; 9 Estación Experimental Agropecuaria Montecarlo, Instituto Nacional de Tecnología Agropecuaria, Misiones N3384, Argentina; 10 Centro de Investigaciones Biotecnológicas del Ecuador, Escuela Superior Politécnica del Litoral, Guayaquil 090112, Ecuador; 11 Departamento de Ciencias Biológicas, Universidad Técnica Particular de Loja, Loja 1101608, Ecuador

**Keywords:** next-generation sequencing, papaya virus X, emerging virus, potexvirus, Argentina

## Abstract

An emerging virus isolated from papaya (*Carica papaya*) crops in northwestern (NW) Argentina was sequenced and characterized using next-generation sequencing. The resulting genome is 6667-nt long and encodes five open reading frames in an arrangement typical of other potexviruses. This virus appears to be a novel member within the genus *Potexvirus*. Blast analysis of *RNA-dependent RNA polymerase* (RdRp) and *coat protein* (CP) genes showed the highest amino acid sequence identity (67% and 71%, respectively) with pitaya virus X. Based on nucleotide sequence similarity and phylogenetic analysis, the name papaya virus X is proposed for this newly characterized potexvirus that was mechanically transmitted to papaya plants causing chlorotic patches and severe mosaic symptoms. Papaya virus X (PapVX) was found only in the NW region of Argentina. This prevalence could be associated with a recent emergence or adaptation of this virus to papaya in NW Argentina.

## 1. Introduction

Papaya (*Carica papaya* L.) is an important fruit crop widely cultivated in tropical and subtropical regions [1]. Papaya ranks fourth among tropical fruits produced worldwide, after banana, mango, and pineapple [2]. The adaptability of this plant and the acceptance of its fruits provide considerable market advantages to this crop. In Argentina, the diversification of tropical fruit species is highly promoted with the aim of contributing to the development of local economies. Papaya trees are grown mainly in Jujuy, Salta, Corrientes, Chaco, Formosa, and Misiones provinces, where the temperature is favorable for its cultivation.

Diseases caused by viruses are the main obstacle to papaya production worldwide [3,4]. Although more than 35 viruses of several taxonomic groups have been reported in tropical and subtropical regions, only papaya ringspot virus (PRSV) and the papaya meleira virus complex are considered threats to papaya production in the Americas. Depending on the time of infection, these viruses can cause complete crop loss rendering the fruit unsellable [5,6,7]. The expansion of agriculture into tropical forests is an important driver of global climate change and biodiversity loss [8]. In this sense, research works have addressed the influence of agricultural activity on climate change and its impact on plant populations, vectors, and the dynamics of different viral pathosystems [9,10]. In the northern region of Argentina, temperature increase may favor the development of tropical crops, by reducing frost damage risk. Nevertheless, the rapid advance of extensive agricultural frontiers in subtropical regions can favor the development of emerging diseases [9].

In northwestern (NW) Argentina, papaya plants showing leaf mottling and mosaic symptoms, which differed from those previously reported for PRSV [11], were detected. In initial analyses of these samples, filamentous virus-like particles were identified and purified from symptomatic leaves. These samples showed cross-reaction in ELISA tests to papaya mosaic virus (PapMV), the only potexvirus reported to naturally infect papaya [12,13], and alternanthera mosaic virus, a potexvirus closely related to babaco mosaic virus (BabMV) that infects babaco (*Vasconcellea* × *heilbornii*) and papaya only by mechanical inoculation [14].

With the development of next-generation sequencing (NGS) technology, plant virus discovery has increased enormously [15] and several emerging papaya viral diseases have been detected in recent years [16,17,18,19]. In this sense, the systematic adoption of high throughput sequencing has contributed to the incremental submissions of sequencing data to public repositories such as NCBI, and the development of platforms for secondary analyses of sequencing data is experiencing a revolution [20,21]. In this study, we characterized the genome sequence of a novel potexvirus infecting papaya plants, according to evolutionary analyses and the demarcation criteria defined for genus *Potexvirus* [13]. This virus represents a new member within this genus. The name papaya virus X (PapVX) is proposed for this new potexvirus, which was not present in any public available global RNAseq library. Symptoms observed in papaya plants were described, and the incidence and geographical distribution of this emergent virus were determined, showing local dispersion in papaya crops from NW Argentina.

## 2. Materials and Methods

### 2.1. Sample Collections and Virus Maintenance

Papaya plants showing leaf mottling and mosaic were first observed in plantations in Yuto, Jujuy, in 2013. Afterward, papaya orchards in NW (Jujuy and Salta) and northeast (NE) (Chaco, Corrientes, Formosa, and Misiones) provinces of Argentina were surveyed from 2017 to 2021. Part of the collected symptomatic and asymptomatic leaf samples was stored at −80 °C, and another part was lyophilized and stored at −20 °C until use. For virus maintenance, leaf samples were ground in phosphate buffer 0.01 M Na_2_HPO_4_, pH 7.0 + 0.1% Na_2_SO_3_, and 600-mesh silicon carbide as abrasive, and the first three expanded leaves were mechanically inoculated onto five healthy papaya cv. ‘Maradol roja’ seedlings with 60 days after germination (dag) of seeds. In addition, five plants were mock-inoculated (control). The inoculated plants were maintained in an insect-free greenhouse at 26 ± 4 °C and exposed to a photoperiod of 14 h light and 10 h darkness, for symptom development and further description [22].

### 2.2. Electron Microscopy

Fresh symptomatic and asymptomatic leaf samples collected from both papaya field plants, and inoculated plants were analyzed by electron microscopy, using leaf-dip and inclusion in Spurr resin technique [23]. Leaf-dips were stained for 1 min with 2% uranyl acetate (Sigma-Aldrich, St. Louis, MO, USA). Moreover, ultrathin sections were double-stained in 0.4% *w*/*v* lead citrate (Sigma Aldrich, St. Louis, MO, USA) and 2% *w*/*v* uranyl acetate. Tissue preparations were examined with a JEM 1200 EX II electron microscope (JEOL, Akishima, Tokyo, Japan) and photographed.

### 2.3. Purification of Virus-like Particles, Library Preparation and Next-Generation Sequencing

**Figure 1:**
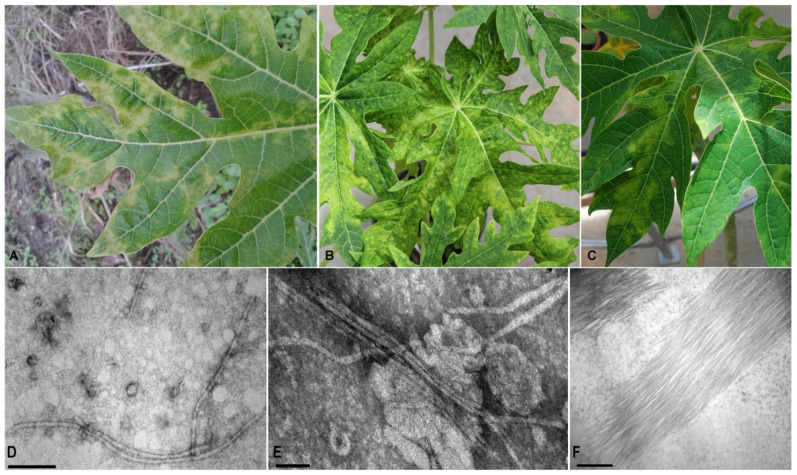
Papaya virus X in Argentina. (**A**) Symptoms (leaf mosaic, mottling, yellowing patches) induced in naturally and (**B**,**C**) mechanically infected papaya plants. (**D**,**E**) Electron micrographs of virus particles in leaf dip of naturally and mechanically infected papaya plants, respectively. (**F**) laminar aggregates in ultrathin sections. D, F-bar = 200 nm; E-bar = 100 nm.

Leaf tissue from one papaya plant showing leaf mottling and mosaic symptoms was used for the purification of virus-like particles (Figure 1A). Plants were collected in 2017 and preserved at −80 °C until use. Plant material was processed using a metagenomics approach based on virion-associated nucleic acids (VANA) [24]. Concentration of virus-like particles was performed according to Lockhart [25], with modifications. Leaves were ground and mixed in a blender with 1:4 (*w*/*v*) of cold 0.05 M Tris-citrate (pH 7.4) containing 0.5% Na_2_SO_3_ (*w*/*v*), 1% polyvinylpyrrolidone (PVP, mol wt 40,000) (*w*/*v*), and 1% Triton X-100 (*w*/*v*). The homogenate was squeezed through muslin and clarified by blending for 20 s; then 25% chloroform (*v*/*v*) was added, and centrifugation was performed at 10,000× *g* for 10 min. The aqueous supernatant was collected, and the virus-like particles were concentrated by centrifugation in a 30% sucrose gradient at 136,000× *g* at 4 °C for 1 h, in a Beckman SW45 Ti rotor. The pellet was resuspended by stirring in 1 mL of 0.05 M Tris-citrate (pH 7.4) containing 0.5% Na_2_SO_3_ (*w*/*v*) at 4 °C for 1 h.

For NGS, viral RNA was extracted from the suspension (200 μL) of concentrated particles using RNeasy Plant Mini Kit (Qiagen, Hilden, Germany), according to the manufacturer’s protocol. RNA quality and concentration were determined by fluorometric quantitation Qubit 2.0 (Thermo Scientific, Waltham, MA, USA). RNA preparation (1 µg), subjected to ribosomal RNA depletion with Ribo-Zero rRNA Removal Kit (Illumina, New York, NY, USA), was used for library construction via TruSeq RNA Library Prep Kit v2 (Illumina, New York, NY, USA), according to the manufacturer’s protocol. The RNA was fragmented and copied into the first-strand cDNA using 200 units of SuperScrip III reverse transcriptase (Invitrogen, Waltham, MA, USA) and random primers; then, the second-strand cDNA was synthesized using 1 unit of DNA polymerase I (Invitrogen, Waltham, MA, USA) and 2 units of RNase H (Thermo Scientific, Waltham, MA, USA), according to the manufacturer’s protocol. The cDNA was purified using Agencourt AMPure XP magnetic beads (Beckman Coulter, Inc., Indianapolis, IN, USA). A total of 3,084,077 paired-end 250-nt long reads were generated using the Illumina MiSeq platform at Instituto de Biotecnología, INTA Castelar, Argentina.

### 2.4. Sequence Processing and De Novo Assembly

A trimming step was performed to remove adapters and low-quality reads using the Trimmomatic software version 0.39 (http://www.usadellab.org/cms/?page=trimmomatic, accessed on 1 December 2017) with standard parameters [26]. The processed reads were de novo assembled using Trinity software version 2.5.0 with standard parameters [27]. The assembled contiguous sequences (contigs) were used as queries for BlastX searching with standard parameters (https://blast.ncbi.nlm.nih.gov/Blast.cgi, accessed on 1 December 2017). The putative viral sequences obtained were curated by interactive reading mapping of filtered reads using the Bowtie 2 version 2.3.2 software (http://bowtie-bio.sourceforge.net/bowtie2/index.shtml, accessed on 1 December 2017) [28]. Genome organization, including identification of genomic features, was performed using the ORF finder available at National Center for Biotechnology Information (NCBI) and motif search tool from detected open reading frames (ORFs) (https://www.ncbi.nlm.nih.gov/orffinder/, accessed from January to February 2018). Structural and functional domains of the detected gene products were predicted using the NCBI-CD-search tool available at https://www.ncbi.nlm.nih.gov/Structure/cdd/wrpsb.cgi (accessed from January to February 2018), against the CDD v3.19 database with an expected value of 0.01. In addition, the InterPro protein signature database was also used (https://www.ebi.ac.uk/interpro/search/sequence/, accessed on 10 October 2022).

### 2.5. Phylogenetic Analysis

For phylogenetic analyses, sequences of Alphaflexiviridae members were obtained from the NCBI database (Table S1). Sequence alignments and comparisons were performed using MUSCLE 3.8.31, with default parameters [29], via SeaView software version 5.04 [30]. To visualize similarity among species, the selected sequences of RdRp were analyzed using the circos method (Circoletto, an online tool to sequence similarity visualization based on Circos) [31]. Moreover, for species demarcation, pairwise identity analyses of gene sequences were performed using the MUSCLE-based pairwise alignment option, implemented in Sequence Demarcation Tool (SDT) version 1.2 [32]. Phylogenetic analyses were performed on complete genome sequences of *CP* and *RdRp* genes for both nucleotide (nt) and amino acid (aa) sequences. Sequence datasets were refined to conserve non-ambiguous sites using GBlocks with the least stringent conditions [33] in SeaView version 4 software. The evolutionary model for each dataset was estimated using the ModelFinder software [34], according to the Bayesian information criterion, selecting TVM+F+R6 (for complete genome), TVM+F+I+G4 (for CP nt), LG+G4 (for CP aa), GTR+F+R7 (for RdRp nt), and LG+F+R6 (for RdRp aa). The phylogenetic tree reconstruction was performed using maximum likelihood method implemented in the IQ-TREE version 1.6.12 software [35], available online (http://iqtree.cibiv.univie.ac.at/) (accessed from August to December 2021) [36]. The SH-like approximate likelihood ratio test (1000 replicates) [37] and ultrafast bootstrap approximation (UFB) (10,000 replicates) [38] were used to evaluate the reliability of branches and groups obtained. The recombination detection program (RDP) 4 package [39] was used to detect possible recombinations across the whole-genome alignment of potexviruses using RDP, GENECONV, BOOTSCAN, MAXIMUM CHI SQUARE, CHIMAERA, SISCAN, and 3SEQ methods, with default parameters.

### 2.6. Host Range Assay

Mechanical inoculations were done by dusting carborundum (600-mesh) on test leaves followed by rubbing infected tissue homogenized in phosphate buffer as previously described (see Sample Collection and Virus Maintenance). Test plants included papaya (60 dag), pitaya (*Hylocereus undatus*), Christmas cactus (*Schlumbergera truncata*), opuntia (*Opuntia* sp.), *Nicotiana glutinosa* (60 dag), and *Nicotiana rustica* (60 dag) (10 plants per species). Plants were maintained in an insect-free greenhouse, as previously described in Section 2.1.

### 2.7. Virus Detection

#### 2.7.1. RT-PCR Detection

To determine the success of virus inoculation, each plant was RT-PCR tested at 60 days post-inoculation. Sense and antisense primers were designed based on the conserved aa motifs in the C-terminal region of the *RdRp* gene (ORF1) of the virus sequence obtained by Illumima MiSeq in this work and reference sequences of other related potexviruses, according to Van der Vlugt and Berendsen [40]. Designed primers were checked using the Primer-Blast tool developed at NCBI (https://www.ncbi.nlm.nih.gov/tools/primer-blast/, accessed on 15 January 2020) and tested on the samples in which this virus was first detected by NGS.

Total RNA was extracted using Trizol method (Thermo Scientific, Waltham, MA, USA), according to the manufacturer’s instructions. The RNA was used for first-strand cDNA synthesis using Moloney murine leukemia virus (MMLV) reverse transcriptase enzyme (Promega, Madison, WI, USA) and random primers (Biodynamics, Buenos Aires, AR). For virus detection, PCR reactions were performed using 4 μL of cDNA, 1.25 U of GoTaq^®^ G2 DNA polymerase enzyme (Promega, Madison, WI, USA), 200 μM dNTPs and 0.3 μM of each primer in a final reaction volume of 50 μL. The PCR conditions included an initial incubation at 95 °C for 2 min, followed by 35 cycles of denaturation at 95 °C for 30 s, annealing at 55 °C for 45 s, extension at 72 °C for 60 s, and a final extension at 72 °C for 10 min.

PCR products were separated on 1.0% agarose gel with TAE buffer (40 mM Tris base, 20 mM acetic acid, 1 mM EDTA pH 8.0). DNA bands were stained with Gel Red (Biotium Inc., San Francisco, CA, USA), using GelDoc EQ (Bio-Rad Laboratories, Hercules, CA, USA) gel imaging system with the Quantity One analysis software version 4.5.1 (Bio-Rad Laboratories, Hercules, CA, USA) and scored by comparison to a 1-kb DNA ladder (PBL, Buenos Aires, Argentina).

#### 2.7.2. Sequence Read Archive (SRA) Searches

To investigate whether the detected virus might be present in other datasets, we employed the Serratus palmID tool [41] which extracts Palmprint sequences using as query the predicted RdRp of this virus with standard parameters. Palmprints are a ~100 aa sub-sequence of the *polymerase* gene containing A, B, and C conserved RdRp motifs [42]. The related SRA datasets were processed as described elsewhere [43]. In addition, the Alphaflexiviridae MSA alignment based on palmprint predictions of Serratus Tree tool was downloaded and a ClustalW consensus alignment with standard parameters was generated including the predicted palmprint of the papaya virus detected. Finally, a rapid FastTree 2.1.11 phylogenetic tree was generated with the obtained alignment.

### 2.8. Virus Distribution and Genetic Analyses

A virus survey was conducted to determine the distribution of this virus in the northern region of Argentina. Samples were collected from papaya orchard and family gardens (sampling sites) located in Jujuy, Salta, Chaco, Corrientes, Formosa, and Misiones provinces. The geographic coordinates for sampling locations were obtained using a handheld global positioning system (GPS) (Table S2). Virus distribution was determined by molecular analysis of symptomatic and asymptomatic leaves previously collected and stored at −80 °C. DIVA-GIS software version 7.5 (http://www.diva-gis.org, accessed on 15 April 2022) was used to generate a map showing the virus distribution data. The virus was also tested in Ecuador, using papaya and babaco. A total of 120 papaya samples were collected from four commercial orchards distributed in three provinces, Santa Elena (n = 30), Manabí (n = 60), and Esmeraldas (n = 30), whereas babaco samples were collected from a commercial plant nursery in Azuay province (n = 30) and a commercial production operation in Pichincha province (n = 30).

The RNA purification, cDNA synthesis, and PCR conditions were the same as previously mentioned. The PCR products corresponding to symptomatic plants, obtained in duplicate, were purified using Wizard^®^ SV Gel and PCR Clean-Up System (Promega, Madison, WI, USA), and then directly sequenced in both directions by Macrogen Inc. The obtained sequences were assembled with Staden package version 2.0.0b10 (https://staden.sourceforge.net, accessed on 1 March 2022), and submitted to the BlastN program [44] for homology searching (http://blast.ncbi.nlm.nih.gov, accessed on 1 March 2022).

After sequence trimming, a 681-bp *RdRp* gene fragment was used to perform the phylogenetic analyses. The phylogenetic tree was also built using the RdRp partial sequences of isolates of the newly proposed papaya virus and other sequences of closely related species. The most related species were chosen according to the grouping obtained in the phylogenetic tree of RdRp complete sequences (Section 2.5). The identity analyses among the obtained sequences were carried out using the MUSCLE-based pairwise alignment option implemented in SDT version 1.2 [32]. Multiple sequence alignments were used for ML phylogenetic reconstruction, as described above. The evolutionary model estimated was TIM3+F+I+G4, according to Bayesian Information Criterion. Papaya samples collected from Argentina were also tested, for two additional potexviruses i.e., PapMV and BabMV, using specific primers [14,45].

## 3. Results

### 3.1. Papaya Symptoms and Particle Observations

Chlorotic patches and severe mosaic symptoms, similar to those observed in the field samples (Figure 1A), were observed in mechanically inoculated papaya plants (Figure 1B,C). Initial symptoms were visible on young leaves at 15 days post-inoculation (dpi), and at 35 dpi the virus was able to systemically infect the inoculated plants. Filamentous virus-like particles (~600 nm) were only observed in leaf dip preparation of symptomatic field samples as well as in mechanically inoculated plants, respectively (Figure 1D,E). Particle aggregates were also observed in ultrathin sections (Figure 1F).

### 3.2. Sequence Assembly

A total of 2,221,356 reads with a mean length of 145-nt were left after trimming and quality control. The processed reads were assembled de novo using Trinity; 124,390 contigs were obtained, which were subsequently used as query for BlastX searches against the NCBI refseq virus proteins. A single 6570-nt long contig obtained a significant hit (67.31% identity, E-value = 0.0) against the RNA-dependent RNA polymerase of pitaya virus X (PiVX). Further inspection of the virus transcript and curation by iterative mapping of filtered reads resulted in a consolidated 6667-nt virus sequence supported by 7564 reads (mean size 108.8-nt), and a mean coverage 131.7X. The virus sequence showing features and identity with several members of the genus *Potexvirus* was further annotated.

### 3.3. Genome Organization and Identification

**Figure 2:**
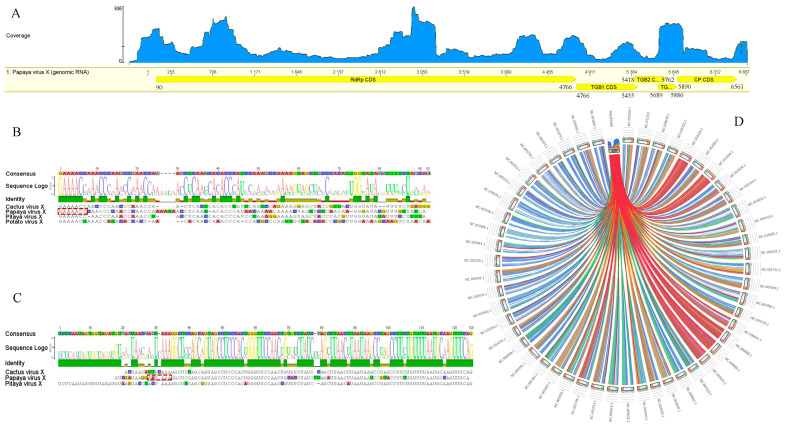
Schematic representation of the genome organization of papaya virus X. (**A**) The large open reading frames (ORF) are depicted by an open box (yellow boxes). The numbers indicate the nucleotide positions of the RNA segments for each mature protein in the polyprotein. A RNAseq-based read mapping track at the top of the PaPVX genome graph represents coverage map per base. (**B**,**C**) Sequence alignments between 5′ and 3′UTRs of PapVX and related potexviruses. The 3′UTR alignments are shown (excluding the polyA tail). (**D**) Sequence similarity plot obtained with Circos. Ribbons represent the local alignments produced by tBlastX, their width the alignment length, and the colors the alignment percent similarity in four quartiles: blue ≤ 58, green ≤ 60, orange ≤ 65, red > 65.

The genome sequence of the detected papaya virus consisted of 6667-nt, excluding a polyA tail, and is organized in five ORFs (Figure 2A). Similar to other potexviruses, the ORF1 (nt 90–4766) encodes the RdRp, consisting of 1558 aa with an estimated molecular weight of 177.6 kDa. The virus possesses a set of three partially overlapping ORFs: ORF2 (nt 4766–5455), ORF3 (nt 5418–5762), and ORF4 (nt 5689–5886), displaying the typical triple gene block (TGB) configuration of potexviruses. ORF5 (nt 5890–6561) encodes the putative coat protein (CP) (Table S3). The conserved aa motifs QDGAML (nt 4155–4172), HQQAKDE (nt 3588–3608), and TFDANTE (nt 4305–4325) were identified in the C-terminal region of the RdRp [40]. Moreover, a catalytic RdRp domain, containing the characteristic core motif TGX3TX3NTX22GDD found in potexviruses [46], was present near the C terminus of the RdRp. The conserved motifs, including viral methyltransferase (Vmethyltransf) [47], RNA helicase (Viral_helicase1) [13], and RdRp [48], were also detected.

Sequence alignments with potexviruses indicated the presence of untranslated regions (UTR) of 89-nt and 106-nt at the 5′ and 3′ ends, respectively. The hexanucleotide 5′-GAAAAC-3′, present at the 5′-UTR ends of most potexviruses, was observed (Figure 2B). A putative polyadenylation signal, 5′-nt 6572 AAUAAA-3′, important for the infectivity of potexviruses [49], was identified downstream of 3′-UTR (Figure 2C). The presence of a typical 5′ end hexanucleotide at the 5′ UTR terminus and a putative polyA signal at the 3′ end indicates that the obtained virus sequence is coding-complete and tentatively corresponds to the complete genome of this novel virus. Similar levels of the RdRp, expressed as Circoletto diagrams, confirmed a major relationship with those members of the genus *Potexvirus* (Figure 2D). A Blast search using RdRp and CP sequences revealed that the detected virus belongs to the genus *Potexvirus*. Pairwise sequence comparisons of the characterized virus and its closest relatives PiVX, zygocactus virus X (ZyVX), cactus virus X (CVX), schlumbergera virus X (SchVX), and opuntia virus X (OpVX) revealed nt identities ranging from 66–68% for the RdRp and 64–67% for the CP. At the amino acids level, an average of 66.50% identity was observed for the RdRp and 66% for the CP (Table S4). In turn, the least conserved *TGB* genes showed nt identities ranging from 62 to 67% for *TGB1*, 56 to 58% for *TGB2*, and 55 to 56% for the *TGB3* (data not shown). Based on the species demarcation criterion for potexviruses, which states that distinct species share <72% (nt) or <80% (aa) identity for the *RdRp* and *CP* genes [10,50], our results (Table S4) strongly support that the detected virus should be considered a new member within this genus, and the name papaya virus X (PapVX) is proposed. The PapVX genome sequence was deposited in Genbank under accession number MN265368.1. The presence of other viruses was not detected in the papaya dataset obtained by NGS.

### 3.4. Exploring Publicly Available Transcriptome Datasets

The tool generates matching data cross-referenced against 5.7-Mb NCBI-SRA sequencing libraries to identify putative SRA-virus matches. The search tool retrieved several virus hits with significant identities to the Palmprint conserved region. The two closest hits (id 89.5%/93.7%, E-value = 3.1 × 10^−61^/2.1 × 10^−68^) corresponded to the NCBI-SRA datasets SRR6846447 of a wastewater metagenome sample from Singapore and SRR11190798 of a pitaya fruit (Hylocereus polyrhizus) sample from Guangdong China, respectively. A rapid NCBI-BlastP of the obtained 95 aa palmprints showed as best hits to the PiVX RdRp (98.95% identity, E-value = 2 × 10^−60^) and to ZyVX RdRp (95.79% id, E-value = 3 × 10^−58^), respectively, suggesting that the retrieved virus-like sequences corresponded to strains of these viruses and certainly not to the PapVX reported here. These two SRA datasets were processed and resulted in the assembly of nearly complete virus genomes (PiVX = 6659-nt, ZyVX = 6483-nt), with the genomic architecture of members of genus *Potexvirus* (view SRA datasets). The encoded complete RdRps of both assemblies were subjected to NCBI-BlastP searches, which retrieved as best hit PiVX isolate GZ-ZYP from China (97.80%, E-value = 0) and ZyVX hosted by *S. truncata* (98.12%, E-value = 0) from Germany for ZyVX. Pairwise identity alignments of PiVX and ZyVX with PapVX showed a genome nt identity of 65.8% and 66.6% respectively and an aa identity of the predicted encoded proteins ranging from 48.7–53.5% for TGB2 protein to the highest of 66.1–70.4% for the CP protein (Figure S1A). The phylogenetic tree generated from obtained alignment, unequivocally showed that PapVX clustered as a separated clade in a subgroup including the PiVX and ZyVX, indicating that both genetic and evolutionary insights suggest that PapVX is a novel member (Figure S1B). Thus, to our knowledge, PapVX appears to be, as of today, a novel undescribed virus that was not present in any publicly available global RNAseq library.

### 3.5. Virus Phylogenetic Analysis

**Figure 3:**
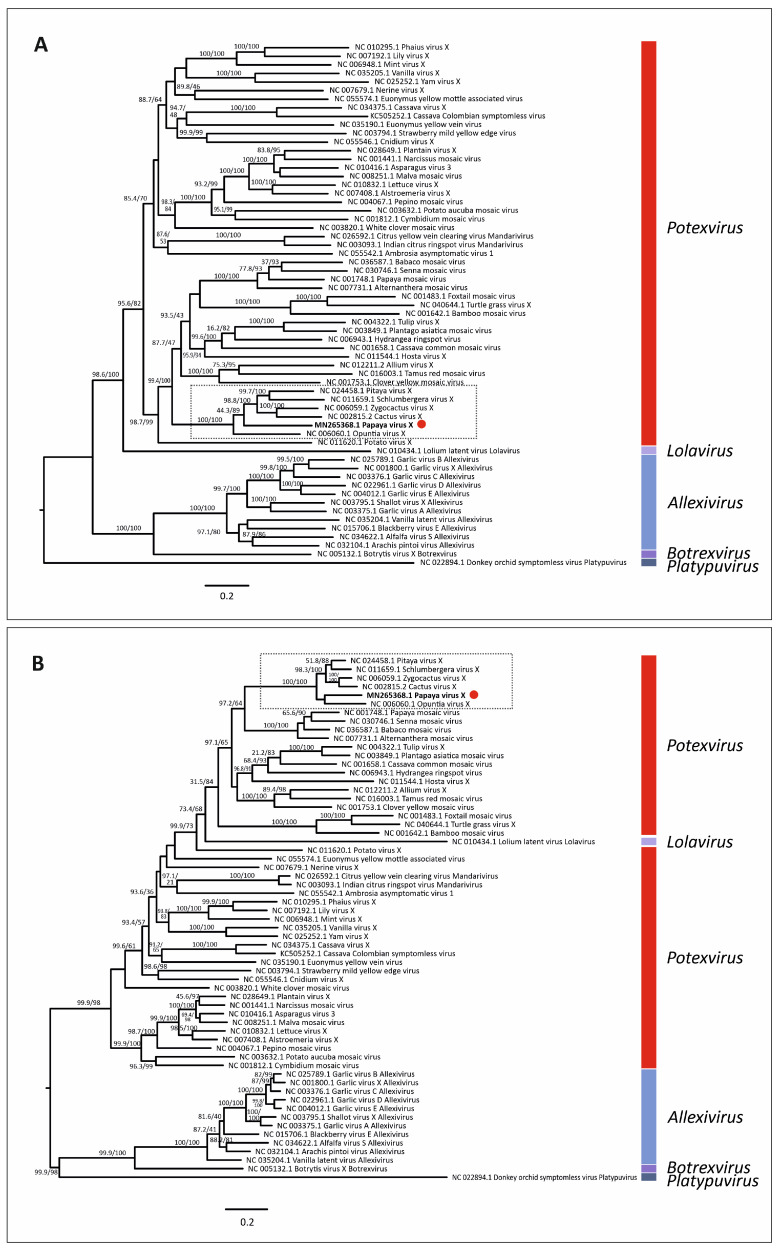
Phylogenetic tree of (**A**) nucleotide and (**B**) amino acid sequences of *replicase* gene of papaya virus X (red dot) and virus species in the family *Alphaflexiviridae*. Branch lengths are proportional to genetic distances (nucleotide and amino acid substitutions per site). The SH-like/UFB support values are indicated when at least one of them is higher than 70%.

**Figure 4:**
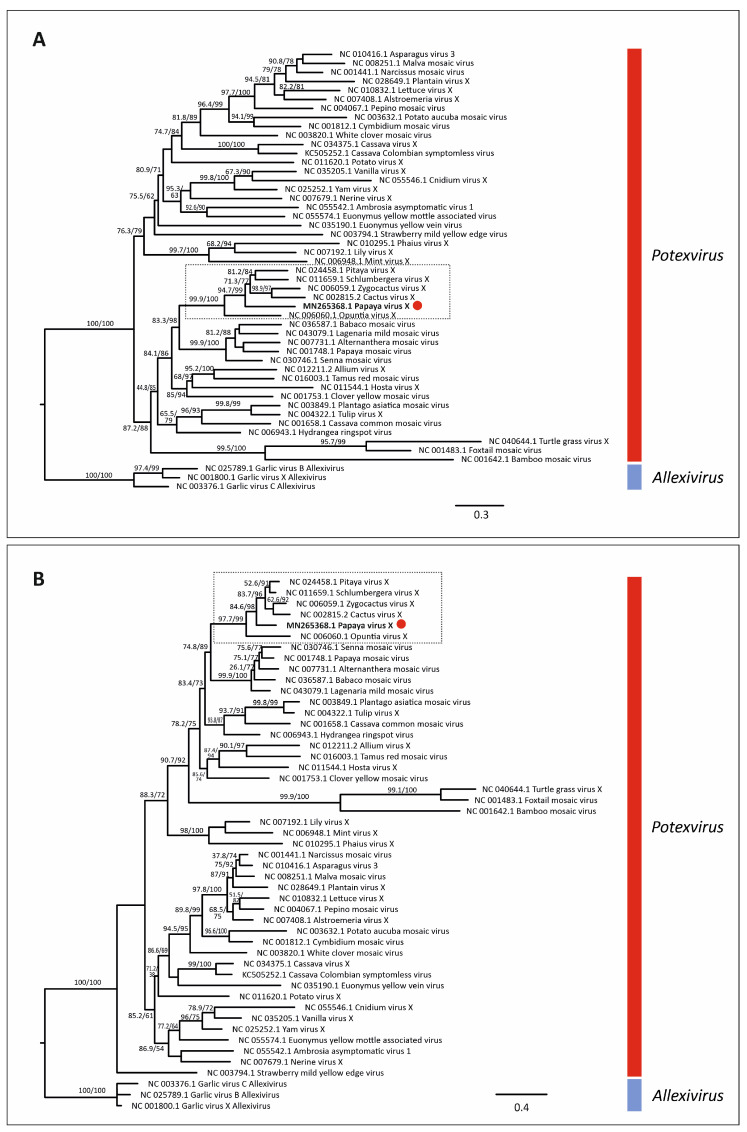
Phylogenetic tree of (**A**) nucleotide and (**B**) amino acid sequences of *coat protein* gene of papaya virus X (red dot) and virus species in the genus *Potexvirus*. Branch lengths are proportional to genetic distances (nucleotide and amino acid substitutions per site). The SH-like/UFB support values are indicated when at least one of them is higher than 70%.

**Figure 5:**
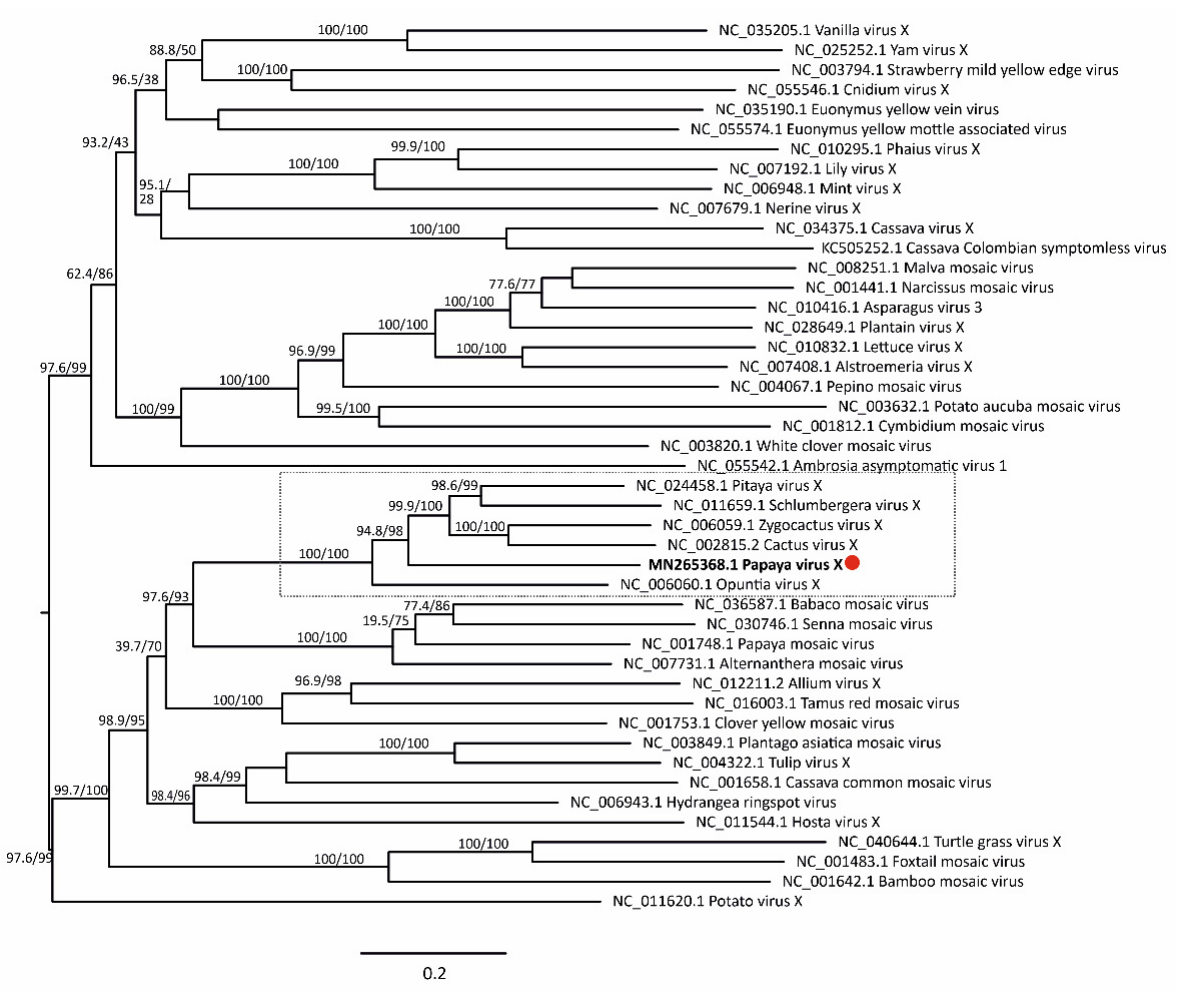
Phylogenetic tree of complete genome sequences of papaya virus X (red dot) and virus species in the genus *Potexvirus*. Branch lengths are proportional to genetic distances (nucleotide substitutions per site). The SH-like/UFB support values are indicated when at least one of them is higher than 70%.

Maximum likelihood phylogenetic trees based on nt and aa sequences of RdRp from 61 alphaflexiviruses showed that PapVX clustered with potexvirus species *pitaya virus X*, *Zygocactus virus X*, *cactus virus X*, *Schlumbergera virus X*, and *Opuntia virus X*, in a highly supported group (Figure 3). The phylogenetic tree based on nt and aa sequences of the *CP* gene of potexviruses (47 species), and genome sequences (nt) (44 species) (Table S4) showed similar grouping among these viruses, with high support values in all trees (Figure 4 and Figure 5). No recombination events were detected among the genome sequences of potexvirus members analyzed.

### 3.6. Host Range Assay and Virus Detection

Back inoculation of PapVX to healthy papaya plants produced mosaic symptoms similar to those previously described. Virus symptoms were not observed in *H. undatus*, *S. truncata*, *Opuntia* sp., *N. glutinosa*, and *N. rustica*. The presence of PapVX was molecularly confirmed only in papaya plants. The developed primer set PapVX5 and PapVX1RC (Table 1) resulted in amplification of ~737-bp PCR products from the terminal region of the *RdRp* gene. The inoculated papaya plants were also tested for PRSV [11], resulting negative for this virus infection.

### 3.7. Virus Distribution and Genetic Analyses

**Figure 6:**
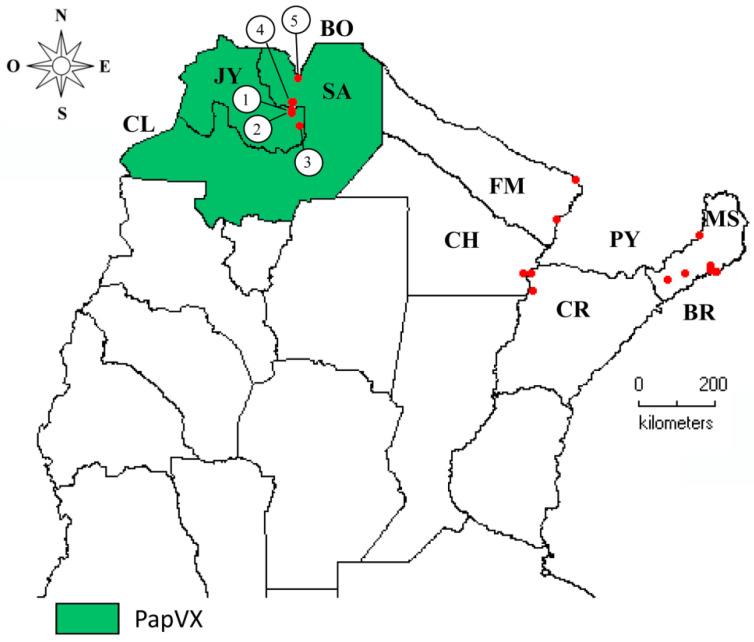
Geographical distribution of papaya virus X infecting papaya crops in Argentina. Locations where viral disease was detected are numbered from 1 to 5, and are shown in Table S2. JY = Jujuy, SA = Salta, FM = Formosa, CH = Chaco, CR = Corrientes, MS = Misiones, CL = Chile, BO = Bolivia; PY = Paraguay, BR = Brazil.

PapVX was identified in 5 (23%) of the 21 localities sampled from the north of Argentina; only numerical tags were assigned to map locations where the virus was identified (Table S2; Figure 6). Of the 36 papaya plantations and family gardens (sampling sites) surveyed (18 from NE and 18 from NW regions), 8 sites were positive for PapVX, showing a prevalence of 22%. This virus was present exclusively in the NW region of Argentina. On the other hand, the potexviruses PapMV and BabMV were not detected in the inspected sites in Argentina. Moreover, PapVX was not detected in any of the papaya or babaco samples tested in Ecuador. PapVX detection primers and RT-PCR conditions were validated using BabMV-infected samples, which were successfully detected in babaco. The sequences obtained in this work were deposited in GenBank; details of the origins are shown in Table S2.

The comparison between the partial *RdRp* gene sequences of PapVX obtained (GenBank accession numbers: ON007250, ON007251, ON007252, ON007253, ON007254) and the MN265368.1 showed nucleotide sequence identity values ranging from 82.99–99.71%, with the lowest identity values being detected between isolates from different locations. The pairwise analysis of the amino acid sequences showed values ranging from 98.56% to 100%. The highest similarities were observed between the sequences of isolates from nearby localities: isolates from Yuto (100%; ON007254–MN265368.1) and Colonia Santa Rosa (100%; ON007251–ON007252). In addition, in the phylogenetic tree of the partial *RdRp* gene sequences of PapVX, two subgroups were observed, one including sequences from Jujuy province (Yuto and Palma Sola) and the other from Salta province (Colonia Santa Rosa and Orán) (Figure S2).

## 4. Discussion

The ICTV has officially recognized 48 species in the genus *Potexvirus* (https://talk.ictvonline.org/taxonomy/, accessed on 10 October 2022). In this work, we report the genetic and epidemiological studies of a novel potexvirus naturally infecting papaya crop in the NW region of Argentina. The sequencing strategy used in this study (TruSeq RNA Illumina protocol) targeted RNA viruses, whereas additional viral metagenomic analyses could help to understand the diversity of viruses infecting papaya in the subtropical region of Argentina.

In this study, the symptoms observed in papaya plants were different from those attributed to PRSV infection, the only viral disease previously reported in this crop in Argentina [11]. Several viruses may cause similar symptoms in one plant species and symptom expression in infected plants could be different according to the viral isolate or viral combinations [51]. In this sense, Mumo et al. [18] reported similar symptoms in papaya plants to those attributed to PRSV infection [4], although PRSV was not detected. The biological methods for virus diagnosis are time-consuming, but they still hold importance in the characterization of plant viruses. In this regard, a clear association between PapVX presence and symptom expression was observed. Interestingly, PapVX was only reported in papaya plants with non-PRSV infection. The possible role of the potexvirus PapMV as a protective agent against PRSV and probably against other viruses was previously suggested [51].

PapVX exhibits close genetic relationships and shares a common ancestor with PiVX, ZyVX, CVX, SchVX, and OpVX; for this reason, the name papaya virus X is proposed. Recombination and mutation events are major forces attributed to evolution in plant viruses and are associated with emergence of new variants and host adaptation [52,53]; however, recombination events were not detected between PapVX and other potexviruses analyzed.

The presence of PapVX only in papaya crops of NW Argentina suggests that the virus has a restricted geographical distribution or that it has been present in papaya and/or other undetected host plants, which remains to be investigated. In this sense, the fact that this virus was detected exclusively in papaya plantations in Salta and Jujuy, but not in the NE region (Corrientes, Chaco, Formosa, and Misiones), suggests a recent emergence in this crop. Even with partial sequences, a geographical clustering was observed, indicating sustained transmission with still limited dispersion.

Although the phylogenetic relationships of PapVX with viruses isolated from cactaceous plants were confirmed, it was not experimentally transmitted to the plant species tested in this family (pitaya, Christmas cactus, and opuntia). In this sense, Alvarez-Quinto et al. [14] showed that genetic diversity among potexviruses is not strictly related to host range. Although mechanical transmission could be a mode of virus dispersal, the successful infection of different plant species by mechanical transmission in the laboratory does not reflect what happens under field conditions. For example, babaco mosaic virus, a babaco-infecting potexvirus in Ecuador, does not infect papaya under field conditions, but mechanical transmission to papaya was successfully achieved [14]. Moreover, no insect vector has been reported to date in PapVX- related viruses (PiVX, ZyVX, CVX, SchVX and OpVX).

The primer set PapVX5 and PapVX1RC resulted in the amplification of a ~737-bp PCR products from the terminal region of the *RdRp* gene and will help to monitor the virus distribution and discover potential new hosts. These primers were employed to analyze papaya and babaco samples from Ecuador; results showed that all samples were negative for PapVX. The prospection of PapVX in papaya orchards from Bolivia, which borders the region where this virus was detected in Argentina, could be important to enhance the knowledge of PapVX dispersion. Linear patterns of papaya plants infected with PapVX were observed in the same crop row, which was potentially associated with mechanical labor (leaf removal) performed in the orchards, showing the importance of implementing cultural measures to avoid dispersion of PapVX within the crop. The potential vertical transmission of PapVX (via seeds), which plays a role in the long-distance dispersion of pathogens [54], should be further characterized.

The use of high-throughput sequencing to detect known and novel plant viruses or variants has extended considerably [55], increasing our understanding of viral complexes and the diseases they cause. Known or emerging plant viruses are often reported in papaya, with more than 35 viral entities being known to cause disease in this crop worldwide. Although most of these viruses have a wide host range, such as tomato spotted wilt orthotospovirus and cucumber mosaic virus, other viruses such as papaya meleira virus and papaya virus Q, have only been detected in papaya [5,56], which could be similar to that observed for PapVX. To unravel these epidemiological aspects associated with the host range, the analysis of native plants associated with the papaya production should be included in the virus prospection.

The tropics and subtropics provide many examples of new encounter situations between viruses and wild or cultivated plants [54,57]. For example, in NW Argentina, several emerging viral diseases have been reported [58,59]. Papaya production in Argentina has increased significantly in the last years; as expected, virus-like diseases have also become conspicuous in the major production areas. The results obtained in this investigation encourage future studies considering the potential of native species as host of plant viruses to provide a complete panorama of virus emergence and evolution in the northern region of Argentina.

## 5. Conclusions

A virus naturally infecting papaya, tentatively named PapVX, is herewith reported for the first time. Given the rate at which papaya planting materials are being exchanged between farmers, it is likely that this virus, although currently restricted to NW of Argentina, could spread to other papaya-growing areas in the region. Additional surveys in other papaya crops areas, including native plants from hotspot sites and neighboring countries, should be carried out to determine the presence of this virus. Moreover, further epidemiological studies (host range, seed transmission) are needed to understand the risk that PapVX poses to papaya and other crops in the region.

## Figures and Tables

**Table 1:** Primers, target region of virus genome, conserved amino acid motifs in the C-terminal region of the viral replicase of potexviruses, and expected amplicon size.

Virus	Genome Region	Position	Amino Acid Motif	Sequence	Amplicon Size (bp)
PapVX	RdRp	1167-1173	HQQAKDE	CACCARCARGCNARRGATGA	737
		1412-1406	TFDANTE	TCDGTGTTKGCRTCRAADGT	

## Data Availability

The complete sequence of papaya virus X isolate Yuto is available at NCBI-GenBank under accession number MN265368. The raw RNAseq sequence *C. papaya* data has been submitted to the NCBI Sequence Read Archive (SRA) under Bioproject PRJNA886645. The partial *RdRp* gene sequences of PapVX isolates are also available (GenBank accession numbers: ON007250, ON007251, ON007252, ON007253, and ON007254).

## Supplementary Materials

The following supporting information can be downloaded at: https://www.mdpi.com/article/10.3390/v14102297/s1. Table S1: List of viruses used for phylogenetic inferences. Table S2: Geographical location of papaya collection sites in Argentina and collection dates. Table S3: Characteristics of deduced proteins encoded by papaya virus X (PapVX) genome determined by predictive algorithms. Table S4: Sequence identities of papaya virus X (MN265368.1) against reference sequences of reported species from genus *Potexvirus*. Figure S1: Surveying the NCBI-SRA database to assess the presence of papaya virus X (PapVX) in publicly available sequencing data. (A) Schematic representation of the genome organization of PapVX, pitaya virus X (PiVX) and zygocactus virus X (ZyVX). Open reading frames (ORF) are depicted by arrowed yellow rectangles. The pairwise nucleotide identity between genomes and amino acid identity based on predicted encoded proteins are showed. (B) Maximum likelihood phylogenetic tree based on Alphaflexiviridae MSA alignment of Serratus palmprint predictions on the NCBI-SRA database and the predicted palmprint of PapVX. The right panel is an inset expanding the cluster of virus serratus predicted palmprints including PapVX. Figure S2: Phylogenetic tree of nucleotide sequences of the replicase gene (partial) of papaya virus X and of the most closely related virus species in the genus *Potexvirus*. Branch lengths are proportional to genetic distances (nucleotide substitutions per site). The SH-like / UFB support values are indicated at nodes and tree is midpoint-rooted. Datasets: Sequence datasets used in the phylogenetic analyses.
