# Supplementary material for: An Unwanted Association: The Threat to Papaya Crops by a Novel Potexvirus in Northwest Argentina
Source: Viruses. 2022 Oct 19;14(10):2297. doi: 10.3390/v14102297 (PMC9610017; doi:10.3390/v14102297)
Supplement: Supplementary file 1 [file viruses-14-02297-s001.zip › Supp Table S1.pdf]

Table S1. List of viruses used for phylogenetic inferences.

Species	NCBI Accession number	Genus
Pitaya virus X	NC_024458.1	*Potexvirus*
Zygocactus virus X	NC_006059.1	*Potexvirus*
Cactus virus X	NC_002815.2	*Potexvirus*
Schlumbergera virus X	NC_011659.1	*Potexvirus*
Opuntia virus X	NC_006060.1	*Potexvirus*
Allium virus X	NC_012211.2	*Potexvirus*
Hosta virus X	NC_011544.1	*Potexvirus*
Hydrangea ringspot virus	NC_006943.1	*Potexvirus*
Tulip virus X	NC_004322.1	*Potexvirus*
Plantago asiatica mosaic virus	NC_003849.1	*Potexvirus*
Cassava common mosaic virus	NC_001658.1	*Potexvirus*
Tamus red mosaic virus	NC_016003.1	*Potexvirus*
Clover yellow mosaic virus	NC_001753.1	*Potexvirus*
Alternanthera mosaic virus	NC_007731.1	*Potexvirus*
Papaya mosaic virus	NC_001748.1	*Potexvirus*
Babaco mosaic virus	NC_036587.1	*Potexvirus*
Senna mosaic virus	NC_030746.1	*Potexvirus*
Vanilla virus X	NC_035205.1	*Potexvirus*
Yam virus X	NC_025252.1	*Potexvirus*
Euonymus yellow vein virus	NC_035190.1	*Potexvirus*
Phaius virus X	NC_010295.1	*Potexvirus*
Lily virus X	NC_007192.1	*Potexvirus*
Mint virus X	NC_006948.1	*Potexvirus*
Cassava virus X	NC_034375.1	*Potexvirus*
Potato virus X	NC_011620.1	*Potexvirus*
Strawberry mild yellow edge virus	NC_003794.1	*Potexvirus*
Plantain virus X	NC_028649.1	*Potexvirus*
Malva mosaic virus	NC_008251.1	*Potexvirus*
Narcissus mosaic virus	NC_001441.1	*Potexvirus*
Asparagus virus 3	NC_010416.1	*Potexvirus*
Lettuce virus X	NC_010832.1	*Potexvirus*
Alstroemeria virus X	NC_007408.1	*Potexvirus*
Cymbidium mosaic virus	NC_001812.1	*Potexvirus*
Pepino mosaic virus	NC_004067.1	*Potexvirus*
Potato aucuba mosaic virus	NC_003632.1	*Potexvirus*
White clover mosaic virus	NC_003820.1	*Potexvirus*
Nerine virus X	NC_007679.1	*Potexvirus*
Euonymus yellow mottle associated virus	NC_055574.1	*Potexvirus*
Cnidium virus X	NC_055546.1	*Potexvirus*
Bamboo mosaic virus	NC_001642.1	*Potexvirus*
Foxtail mosaic virus	NC_001483.1	*Potexvirus*
Turtle grass virus X	NC_040644.1	*Potexvirus*
Ambrosia asymptomatic virus 1	NC_055542.1	*Potexvirus*

Cassava Colombian symptomless virus	KC505252.1	*Potexvirus*
Lagenaria mild mosaic virus	NC_043079.1	*Potexvirus*
Indian citrus ringspot virus	NC_003093.1	*Potexvirus*
Citrus yellow vein clearing virus	NC_026592.1	*Potexvirus*
Vanilla latent virus	NC_035204.1	*Allexivirus*
Alfalfa virus S	NC_034622.1	*Allexivirus*
Arachis pintoi virus	NC_032104.1	*Allexivirus*
Garlic virus B	NC_025789.1	*Allexivirus*
Garlic virus D	NC_022961.1	*Allexivirus*
Blackberry virus E	NC_015706.1	*Allexivirus*
Garlic virus X	NC_001800.1	*Allexivirus*
Garlic virus E	NC_004012.1	*Allexivirus*
Shallot virus X	NC_003795.1	*Allexivirus*
Garlic virus A	NC_003375.1	*Allexivirus*
Garlic virus C	NC_003376. 1	*Allexivirus*
Lolium latent virus	NC_010434.1	*Lolavirus*
Botrytis virus X	NC_005132.1	*Botrexvirus*
Donkey orchid symptomless virus	NC_022894.1	*Platypuvirus*
