# Supplementary material for: An Unwanted Association: The Threat to Papaya Crops by a Novel Potexvirus in Northwest Argentina
Source: Viruses. 2022 Oct 19;14(10):2297. doi: 10.3390/v14102297 (PMC9610017; doi:10.3390/v14102297)
Supplement: Supplementary file 1 [file viruses-14-02297-s001.zip › Supp Table S2.pdf]

Table S2. Geographical location of papaya collection sites in Argentina and collection dates.

Province	Department	Location	Date	GPS data	Map[a]
Jujuy	Ledesma	Yuto	2013	-23.639104, -64.475260	1
Jujuy	Ledesma	EEA INTA Yuto	2017	-23.586567, -64.507023	2
Jujuy	Santa Bárbara	Palma Sola	2018	-23.970503, -64.294820	3
Jujuy	Ledesma	Paraje Río Piedras	2018	-23.401801, -64,506167	
Salta	Orán	Colonia Santa Rosa	2018	-23.401801, -64.452116	4
Salta	Orán	Peña Colorada	2018	-22.811360, -64.350422	5
			2018	-22.812280, -64.351546	
Chaco	San Fernando	Resistencia	2017	-27.468875, -58.974200	
Formosa	Formosa	Formosa	2019	-26.192428, -58.179419	
Formosa	Pilcomayo	Clorinda	2013	-25.251871, -57.728898	
Corrientes	Corrientes	Corrientes	2013	-27.475492, -58.777925	
Corrientes	Corrientes	Empedrado	2021	-27.903315, -58.737316	
Misiones	Leandro N. Alem	Cerro Azul	2015	-27.640641, -55.504605	
Misiones	Oberá	Oberá	2016	-27.491442, -55.114986	
Misiones	25 de Mayo	Paraje El Palmital, Colonia Aurora	2019	-27.286206, -54.489858	
Misiones	25 de Mayo	Paraje Cerro Grande, Colonia Aurora	2019	-27.407544, -54.489872	
Misiones	25 de Mayo	Paraje Las Limas, Colonia Aurora	2019	-27.326556, -54.353164	
Misiones	25 de Mayo	Paraje El Progreso, Colonia Aurora	2019	-27.390458, -54.454375	
Misiones	25 de Mayo	Paraje Alicia Baja, Colonia Aurora	2019	-27.444689, -54.367283	
Misiones	25 de Mayo	Paraje Alicia Baja, Colonia Aurora	2019	-27.429650, -54.357339	
Misiones	25 de Mayo	Paraje Alicia Baja, Colonia Aurora	2019	-27.453594, -54.339841	
Misiones	Montecarlo	Montecarlo	2019	-26.566604, -54.759699	

[a] Map references used in Figure 6.
