# Supplementary material for: An Unwanted Association: The Threat to Papaya Crops by a Novel Potexvirus in Northwest Argentina
Source: Viruses. 2022 Oct 19;14(10):2297. doi: 10.3390/v14102297 (PMC9610017; doi:10.3390/v14102297)
Supplement: Supplementary file 1 [file viruses-14-02297-s001.zip › Supp Table S3.pdf]

Table S3. Characteristics of deduced proteins encoded by papaya virus X (PapVX) genome determined by predictive algorithms.

ORF No[a]	Gene	Calculated *Mr* (kDa)	Highest scoring virus protein/*E*-value/query coverage (Blast P)
1	*RdRp*	1503.182	PiVX/0.0/100%
2	*TGB1*	220.555	PiVX/3 x 10^{-113}/97%
3	*TGB2*	108.636	CVX/6 x 10^{-34}/98%
4	*TGB3*	62.273	PVX/5 x 10^{-15}/96%
5	*CP*	214.090	SchVX/3 x 10^{-109}/100%

[a] ORF numbers are represented in the 5' to 3' direction of the viral genome and correspond to those in Figure 3A.
