# Supplementary material for: An Unwanted Association: The Threat to Papaya Crops by a Novel Potexvirus in Northwest Argentina
Source: Viruses. 2022 Oct 19;14(10):2297. doi: 10.3390/v14102297 (PMC9610017; doi:10.3390/v14102297)
Supplement: Supplementary file 1 [file viruses-14-02297-s001.zip › Supp Table S4.pdf]

Table S4. Sequence identities of papaya virus X (MN265368.1) against reference sequences of reported species from *Potexvirus* genus.

**Species**	**Whole genome** (nt)	**ORF1 RdRp** (nt/aa)	**ORF5 CP** (nt/aa)
Pitaya virus X	68.01	67.94/67.85	67.57/69.51
Zygocactus virus X	67.66	68.41/67.30	65.57/66.37
Cactus virus X	66.65	67.02/65.04	64.98/66.36
Schlumbergera virus X	66.26	66.94/67.81	66.97/69.96
Opuntia virus X	66.19	66.88/64.50	64.55/58.11
Allium virus X	60.05	60.87/ 48.36	57.21/36.77
Hosta virus X	60.39	61.49/44.89	55.66/36.11
Hydrangea ringspot virus	61.42	63.19/52.55	57.66/49.55
Tulip virus X	61.19	62.02/50.71	58.88/42.93
Plantago asiatica mosaic virus	61.15	62.56/49.89	58.19/43.07
Cassava common mosaic virus	59.98	60.55/49.12	57.84/ 45.29
Tamus red mosaic virus	59.07	59.53/46.34	57.96/42.73
Clover yellow mosaic virus	60.58	60.93/48.13	58.33/43.54
Alternanthera mosaic virus	59.60	60.46/50.47	60.47/46.34
Papaya mosaic virus	60.32	61.34/51.55	59.54/44.13
Babaco mosaic virus	59.39	60.05/50.46	57.12/44.65
Senna mosaic virus	59.84	60.92/51.78	57.07/45.32
Vanilla virus X	59.23	59.86/44.78	54.24/36.57
Yam virus X	59.83	59.52/45.67	62.50/38.79
Euonymus yellow vein virus	59.75	59.38/44.08	56.97/31.53
Phaius virus X	59.83	60.59/48.25	57.57/37.86
Lily virus X	60.08	61.23/47.48	56.02/37.0
Mint virus X	60.69	60.69/47.19	57.07/33.79
Cassava virus X	60.54	60.54/47.21	59.32/33.18
Potato virus X	58.64	59.28/48.52	57.64/38.12
Strawberry mild yellow edge virus	58.41	59.48/48.15	56.45/32.57
Plantain virus X	58.50	59.21/41.79	57.03/28.05
Malva mosaic virus	58.50	58.64/42.50	60.71/30.77
Narcissus mosaic virus	58.43	59.11/41.66	57.28/29.22
Asparagus virus 3	59.40	59.97/43.95	56.18/30.59
Lettuce virus X	59.82	60.87/43.07	57.39/32.74
Alstroemeria virus X	58.40	58.67/44.53	58.68/32.58
Cymbidium mosaic virus	59.03	60.75/44.79	54.10/33.95
Pepino mosaic virus	58.34	59.04/43.59	59.62/32.42
Potato aucuba mosaic virus	58.31	58.63/41.13	56.76/34.58
White clover mosaic virus	59.66	60.63/50.51	58.26/36.70
Nerine virus X	58.65	59.69/45.20	57.62/33.94
Euonymus yellow mottle associated virus	60.33	60.37/47.82	60.55/35.16
Cnidium virus X	60.03	60.09/47.71	56.15/30.23
Bamboo mosaic virus	59.09	60.34/47.22	58.28/28.90
Foxtail mosaic virus	59.09	60.23/47.87	56.59/26.19

Turtle grass virus X	59.16	59.62/46.78	56.45/22.43
Ambrosia asymptomatic virus 1	59.26	59.46/45.19	57.24/38.53
Cassava Colombian symptomless virus	60.63	61.87/46.59	58.40/34.09
Lagenaria mild mosaic virus	-	-	57.26/47.09
**Threshold[a]**	**-**	**72/80**	**72/80**

[a] The thresholds are as defined by Adams et al. (2004) and Kreuze et al. (2020). The thresholds currently accepted by the ICTV as criteria for species demarcation are indicated in bold. ORF: open reading frame; RdRp: RNA-dependent RNA polymerase; CP: coat protein.
