# Supplementary figures and images for: An Unwanted Association: The Threat to Papaya Crops by a Novel Potexvirus in Northwest Argentina
Source: Viruses. 2022 Oct 19;14(10):2297. doi: 10.3390/v14102297 (PMC9610017; doi:10.3390/v14102297)

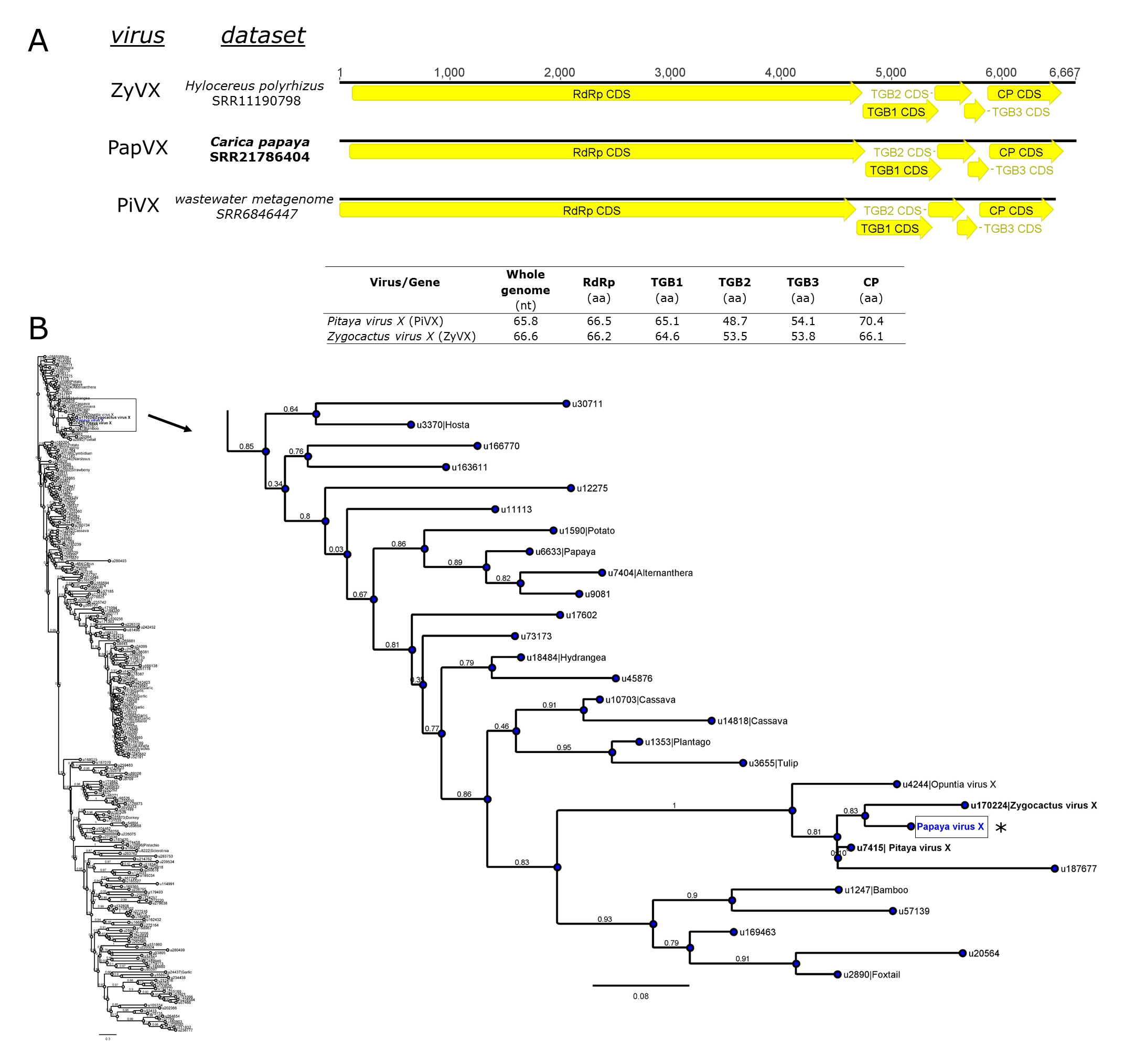

Supplement: Supplementary file 1 [file viruses-14-02297-s001.zip › Supp Figure S1.tif]

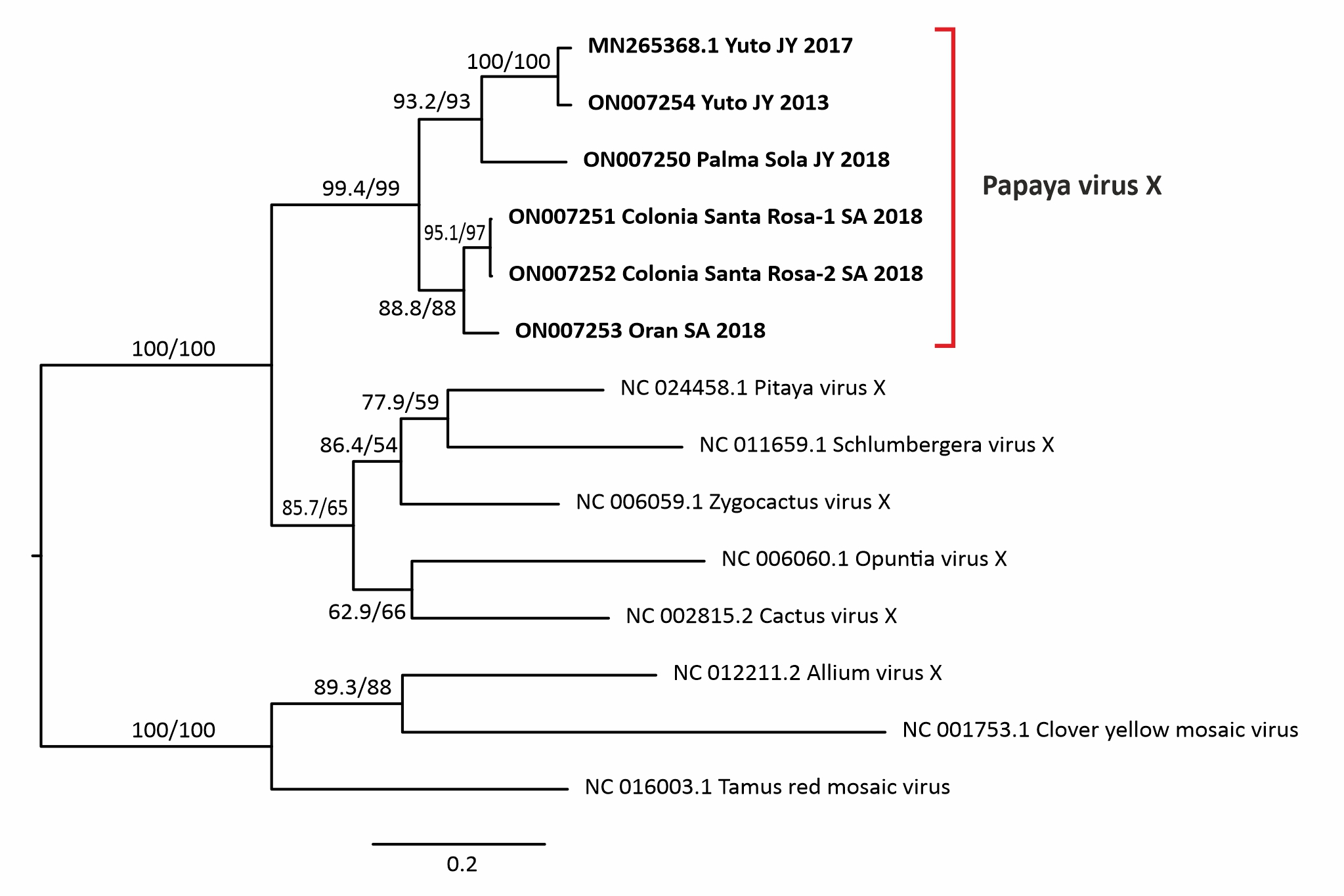

Supplement: Supplementary file 1 [file viruses-14-02297-s001.zip › Supp Figure S2.jpg]
